# Rheumatoid Arthritis and Swine Influenza Vaccine: A Case Report

**DOI:** 10.1155/2012/785028

**Published:** 2012-07-03

**Authors:** Gurjot Basra, Praveen Jajoria, Emilio Gonzalez

**Affiliations:** ^1^Department of Internal Medicine, University of Texas Medical Branch, Galveston, TX 77555, USA; ^2^Division of Rheumatology, University of Texas Medical Branch, Galveston, TX 77555, USA

## Abstract

Rheumatoid arthritis (RA) is the most common chronic inflammatory joint disease. Multiple scientific articles have documented that vaccinations for influenza, MMR, and HBV, to name a few, could be triggers of RA in genetically predisposed individuals. However, there is limited data regarding the association of swine flu vaccine (H1N1) and RA. We report the case of a Mexican American female who developed RA right after vaccination with H1N1 vaccine. Genetically, RA has consistently been associated with an epitope in the third hypervariable region of the HLA-DR *β* chains, known as the “shared epitope”, which is found primarily in DR4 and DR1 regions. The presence of HLA-DRB1 alleles is associated with susceptibility to RA in Mexican Americans. Hence, certain individuals with the presence of the “shared epitope” may develop RA following specific vaccinations. To our knowledge, this is the first reported case of RA following vaccination with the swine flu vaccine.

## 1. Introduction

Rheumatoid arthritis (RA) is the most frequent of all chronic inflammatory joint diseases characterized by pain, swelling, stiffness, and destruction of joints due to synovial inflammation and effusion resulting in disability. It affects 0.5–1% of population in industrialized world with annual incidence reported to be around 12–1200 per 100,000 [1]. Women are affected two to three times as often as men and peak age of onset is between the ages 30 and 55 but can occur at any age. The etiology of RA is multifactorial and includes hormonal, environmental, genetic, infectious, and other variables. RA is associated with the presence of an epitope in the third hypervariable region of the HLA-DR *β* chains, known as the “shared epitope." Individuals with the sequence leu-glu-lys-arg-ala in residues 67–74 have a much higher incidence of developing RA [2]. This sequence is found in DR4, DR14, and DR1 *β* chains. HLA-DRB1 allele is associated with susceptibility to RA in Mexican Americans while HLA-DRB1∗08 appears to have a protective influence on RA susceptibility and disease severity in Mexican Americans.

It has been shown that the onset of rheumatic disease after vaccination signifies that the vaccine may trigger persistent autoimmune response in genetically predisposed individuals. Vaccinations that are suspected to cause RA include influenza, MMR, HBV, tetanus toxoid, typhoid, paratyphoid A and B (TAB), polio, diphtheria, and small pox [3]. HLA DR-4 was frequently present in the above-described patients, suggesting that genetically susceptible individuals are at increased risk for RA after vaccinations. However, there is no causal association between swine flu vaccination and RA onset that has been documented so far. We report a case of Mexican American female who develops RA after vaccination (H1N1) exposure which could possibly be due to her genetic susceptibly explained by above factors. 

## 2. Case Report

A 33-year-old Hispanic female with no significant past medical history was referred to the rheumatology clinic in May 2010 by her primary care physician for having pain and swelling in multiple joints since November 2009. Patient received a seasonal flu shot in October and a week later, she noted some joint pain and aches in hands, wrists, and knees, which resolved within a few days and patient became asymptomatic. A month later, she received H1N1 (swine influenza) vaccine and a week after started experiencing joint pain, swelling, and stiffness in hand, wrist, elbow, shoulder, and knee joints. She also complained of significant morning stiffness that last for more than one hour. On physical examination she had bilateral synovitis in fourth and fifth metacarpophalangeal (MCP), second, and third proximal interphalangeal (PIP) joints, with prominent left ulnar styloid. She fulfilled most of the criteria for classification of RA based on American College of Rheumatology (ACR) guidelines.

Laboratory findings initially included an erythrocyte sedimentation rate (ESR) of 24 mm/hr, rheumatoid factor (RF) of 26 IU/mL, C-reactive protein (CRP) of 1.3 mg/dL, cyclic citrullinated peptide (CCP) IgG of 212. Her basic chemistry, liver function tests, hemoglobin, and complement levels were essentially within normal limits. Serological tests were positive for antinuclear antibody (ANA), RF, anticyclic citrullinated peptide (anti-CCP) and negative for SSA, SSB, hepatitis C virus, parvovirus B19 and being immunized for hepatitis B. Her radiological findings were negative for any joint effusion or destruction. On initial presentation to her PCP, she was given a 10-day course of prednisone 40 mg daily, which partially helped with symptoms in the beginning with eventual relapse of symptoms. Later she was started on methotrexate 7.5 mg weekly along with a short duration (3 weeks) of low-dose prednisone with daily folic acid and calcium/vitamin D supplementation. During her follow-up visit about 2 months later, her disease activity score (DAS) were 4.83 and number of tender joints was 7 and number of swollen joints were 5, with significant improvement in morning stiffness which lasted for 5–10 minutes. However, her pain was controlled only while she was on steroid therapy for 3 weeks and had returned thereafter. Later she was switched to golimumab (TNF-*α* inhibitor) 50 mg once monthly subcutaneously. Her methotrexate dose was also increased to 12.5 mg weekly. At her third visit her symptoms were much improved and her ESR and CRP were also within normal limits. 

## 3. Discussion

Seasonal influenza vaccine has been documented to cause many rheumatic complications, such as, reactive arthritis, polymyalgia rheumatica, and polyarteritis nodosum (PAN), but there is very little evidence in the literature for any association between swine influenza vaccination and RA. However, in 1976, 44 million adults in the USA were immunized with swine flu vaccine. Subsequently many claims were filed against the USA government for various rheumatological adverse reactions including RA. Kurland et al. looked at the incidence in some populations in the few months before and after the immunization program and found them to be no different, hence showing a lack of association between swine flu vaccine and RA [4]. However, in our case report, there is a temporal relationship between swine influenza vaccination and onset of RA. There might also be a synergetic effect of seasonal flu and swine flu vaccines in our study. This patient could be genetically susceptible since she is a Mexican American.

Vaccine-triggered autoimmune reactions can involve two different processes: “antigen specific” in which vaccine products share epitope mimicry and “antigen nonspecific” in which the vaccine activates autoreactive T cells that release cytokines. Vaccine led major histocompatibility complex (MHC) class II induction could facilitate the presentation of autoantigens and the activation of autoreactive T cells, initiating a cascade of self-propagating autoimmunity. Vaccines may contain potentially noxious substances and trace contaminants. Besides antigens and contaminants, adjuvants could stimulate immune responses nonspecifically acting through Toll-like receptors to induce interferon-alpha and the effective processing of self-antigens [5].

It has been documented by Ferrazzi et al. that some of the patients who developed RA or SLE after vaccination were carriers for HLA-DR1 or HLA DR 4/ HLA B27 [6]. Therefore, if there were a screening module available for these genetically predisposed individuals prior to immunization, it would decrease the incidence and morbidity/disability in these patients. There is also a concern regarding vaccination use in a patient with documented RA, since vaccine may trigger RA in susceptible individuals. Review of the literature done by Fabrizio et al. documented that it is safe to use influenza and pneumococcal vaccines in RA [7]. In conclusion, a causal relationship has not been confirmed between swine-flu vaccine and onset of RA, but there is a temporal connection between the two based on our case report.
